# Noninvasive Identification of Isocitrate Dehydrogenase 1/2 Mutation in Brainstem Gliomas

**DOI:** 10.1200/PO-25-00859

**Published:** 2026-02-25

**Authors:** Marina Kushnirsky, Andrew L. Lin, Tejus Bale, Nelson S. Moss, Marc Rosenblum, Sameer Farouk Sait, Lauren R. Schaff, Igor T. Gavrilovic, Elena Pentsova, Christian Grommes, Alicia Meng, Brandon Imber, Viviane Tabar, Kenny Kwok Hei Yu, Alexandra Miller, Rachna Malani, Matthias A. Karajannis, Ingo Mellinghoff, Robert J. Young, Sunitha Thakur

**Affiliations:** ^1^ Department of Neurology, Memorial Sloan Kettering Cancer Center, New York, NY; ^2^ Department of Neurosurgery, Memorial Sloan Kettering Cancer Center, New York, NY; ^3^ Weill Cornell Medical College, New York, NY; ^4^ Department of Pathology, Memorial Sloan Kettering Cancer Center, New York, NY; ^5^ Department of Pediatrics, Memorial Sloan Kettering Cancer Center, New York, NY; ^6^ Department of Radiology, Memorial Sloan Kettering Cancer Center, New York, NY; ^7^ Department of Radiation Oncology, Memorial Sloan Kettering Cancer Center, New York, NY; ^8^ Department of Medical Physics, Memorial Sloan Kettering Cancer Center, New York, NY

## Abstract

**PURPOSE:**

A subset of brainstem gliomas harbor an *isocitrate dehydrogenase 1/2* (*IDH1/2*) mutation, which has important prognostic and treatment implications. We evaluated the radiographic features and the sensitivity of magnetic resonance spectroscopy (MRS) and cerebrospinal fluid cell-free DNA (CSF cfDNA) sequencing at detecting *IDH1/2* mutations in a cohort of these tumors.

**METHODS:**

We identified IDH-mutant brainstem gliomas by retrospective chart review. *IDH1/2* mutation was established by biopsy, CSF cfDNA sequencing, and/or the presence of a 2-hydroxyglutarate (2HG) peak by 3-Tesla MRS.

**RESULTS:**

Twenty-one patients with IDH-mutant brainstem gliomas were identified, 57% male with a median age of 26 (6-59) years. All tumors involved the pons and/or abutted the brachium pontis. Although 18 (86%) of 21 were nonenhancing, only one of 21 demonstrated T2-FLAIR mismatch. An *IDH1/2* mutation was identified by biopsy in 17 and by CSF cfDNA sequencing in three; in the final patient, an *IDH1/2* mutation was inferred by the presence of a 2HG peak. The sensitivity of MRS at identifying 2HG in patients with confirmed *IDH1/2* mutations was 64.3% (95% CI, 35.1 to 87.2), which increased to 85.7% (95% CI, 42.1 to 99.6) when the study was performed before treatment. The 2HG peak was absent in all four patients with a repeat MRS after completion of radiation. *IDH1/2* mutation was identified in four of seven patients who underwent sequencing of CSF cfDNA. In this cohort, median progression-free survival and overall survival were 57.6 and 90.4 months, respectively. An objective response to radiotherapy was observed in 76%.

**CONCLUSION:**

IDH-mutant brainstem gliomas have a characteristic clinical and radiographic phenotype. MRS is more sensitive than CSF cfDNA sequencing for noninvasively identifying the presence of an *IDH1/2* mutation when performed before radiotherapy.

## INTRODUCTION

Brainstem gliomas represent about 4.3% of malignant primary brain tumors in adults and 10%-20% of central nervous system tumors in children.^1,2^ Approximately 15% of brainstem gliomas harbor gain-of-function mutations in *isocitrate dehydrogenase 1* or *2* (*IDH1/2*).^3^ Hierarchical clustering of methylation profiles reveals that infratentorial IDH-mutant astrocytomas cluster separately from supratentorial IDH-mutant astrocytomas and oligodendrogliomas across age categories, suggesting that they comprise a distinct tumor subtype.^4^

**CONTEXT**

**Key Objective**
To evaluate the radiographic features of isocitrate dehydrogenase (IDH)–mutant brainstem glioma and the sensitivity of magnetic resonance spectroscopy (MRS) and cerebrospinal fluid cell-free DNA (CSF cfDNA) sequencing at identifying brainstem gliomas with an *IDH1/2* mutation.
**Knowledge Generated**
IDH-mutant brainstem gliomas involve the pons and/or abut the brachium pontis, are frequently nonenhancing, and rarely exhibit T2-FLAIR mismatch. MRS detects 2-hydroxyglutarate with high sensitivity when performed on IDH-mutant brainstem gliomas before radiotherapy. Although less sensitive, CSF cfDNA sequencing is valuable as confirmatory testing.
**Relevance**
For patients with brainstem glioma, MRS and CSF cfDNA testing are useful for prognostication and clinical management.

IDH-mutant brainstem gliomas have distinct radiographic characteristics, histopathology, and prognosis compared with other brainstem tumors including diffuse midline glioma, H3-K27M altered^5-13^ and adult IDH-wildtype brainstem gliomas.^14,15^ Identification of an *IDH1/2* mutation is prognostically and clinically important, as IDH-mutant brainstem gliomas have a better survival^4,9,15-18^ and are sensitive to chemotherapy (single-agent temozolomide [TMZ] and procarbazine/lomustine/vincristine)^19,20^ and the IDH inhibitor, vorasidenib.^21^

Gain-of-function mutations in *IDH1/2* result in the accumulation of 2-hydroxyglutarate (2HG), an oncometabolite detectible by magnetic resonance spectroscopy (MRS).^22^ Among diffuse gliomas, *IDH1* mutations are more common, with *IDH1* R132H accounting for over 90%.^10,11,13,23,24^ The less common, noncanonical *IDH1/2* mutations, including *IDH1* R132C/S/G/L and *IDH2* R172K/M/W, generate more 2HG than *IDH1* R132H mutations^25^ and are enriched at variable rates in infratentorial IDH-mutant disease,^4,13^ with potential prognostic implications.^4,26^

The brainstem is a compact structure serving critical functions; consequently, damage to a small area may result in significant morbidity. Nevertheless, the risk of stereotactic needle biopsy of the brainstem is generally acceptable due to the therapeutic importance of a tissue diagnosis.^13,27^ Because brainstem biopsies are higher risk and are more prone to undersampling,^13,27,28^ additional strategies are needed to identify *IDH1/2* mutations, either as an adjunct or as a surrogate because of patient preference or clinical circumstance.

MRS is a noninvasive method that identifies IDH-mutant supratentorial tumors by detecting 2HG.^22^ A positive 2HG peak, indicating an *IDH1/2* mutation, was found in 91% of patients when the MRS voxel volume is ≥8 mL and in 42% of patients with MRS volume of >3.4 mL and <8 mL.^22^ MRS has been evaluated in IDH-mutant brainstem gliomas and found to be highly sensitive.^11^ Evaluation of cerebrospinal fluid cell-free DNA (CSF cfDNA), which detects tumor-derived mutations in approximately 50% of patients,^29^ is another noninvasive method. This study evaluates the performance of MRS in a single-center cohort of patients who underwent biopsy and sequencing of CSF cfDNA.

## METHODS

### Patient Population and Clinical Details

A waiver of informed consent was obtained from the Institutional Review Board of Memorial Sloan Kettering Cancer Center. IDH-mutant brainstem gliomas were identified via institutional and departmental databases. Patients were included if they had a positive 2HG peak on MRS, positive CSF cfDNA, and/or biopsy-proven *IDH1/2* mutation. All patients who underwent MRS between June 2013 and September 2023 were reviewed (n = 304), 22 of whom had infratentorial tumors. Subsequently, a neuropathology database was queried using the search terms: brainstem, cerebellar, pontine, pons, medulla, medullary, and midbrain from January 2015 to September 2023 (n = 2,947). These patients were reviewed, and those with a biopsy-proven IDH-mutant brainstem glioma were included (n = 17). Finally, a database of patients who underwent sequencing of CSF cfDNA was reviewed for patients with a posterior fossa tumor and an *IDH1/2* mutation (n = 4).

Chart review was performed on this cohort. Clinical details collected included demographic information (age, ethnicity, gender), histopathologic and sequencing data, treatment response, and survival. Statistical analysis was conducted using Prism (GraphPad Software; Boston, MA). Tumor growth and treatment responses were analyzed using the Response Assessment in Neuro-Oncology for Low-Grade Gliomas criteria.^30^ Progression-free survival and overall survival (OS) were calculated using the Kaplan-Meier method. Survival was calculated from the time of radiographic diagnosis. 95% CIs were calculated using the Clopper-Pearson Exact method.

### Magnetic Resonance Imaging and Spectroscopy

MR studies were conducted using a 3T clinical scanner (MR750, GE HealthCare Technologies; Waukesha, WI) equipped with an 8-channel phased-array head coil. The clinical MR studies were acquired per the standardized brain tumor imaging protocol consensus recommendations.^31^

Single voxel acquisition was performed for 2HG-MRS, using an asymmetric spin echo point-resolved spectroscopy (PRESS) sequence. Subecho times of 26 ms (TE1) and 71 ms (TE2) with a total echo time (TE) of 97 ms were used with a repetition time of 2 seconds. Shaped radiofrequency pulses were used for both excitation (3.6 ms pulse duration with a 90° flip angle) and refocusing (5.2 ms pulse widths with 137° reduced flip angles). Spectral width was 5,000 Hz and 4,096 sampling data points. Averages were 128 for MRS volumes ≥8 mL and 256 for volumes ≤3.4 mL. Reference scans without water suppression were also collected with 16 averages. T2/FLAIR images were used for voxel placement. Total scanning time for MRS, including prescan adjustments for shimming and water suppression, was <5-10 minutes. The unsuppressed water linewidth during prescan was <5-6 Hz full width at half maximum (FWHM). Chemical shift selective pulses were applied for water saturation during PRESS acquisition, and outer volume saturation pulses were used to avoid any contamination from tissue outside of the MRS volume. The MRS scan is suitable for quantitative 2HG analysis only when the spectral peak signal-to-noise ratio is ≥5 and FWHM is ≤8 Hz.

MRS raw data were preprocessed for frequency and eddy current correction, as previously described.^22^ These data were used as input to perform metabolite quantification by spectral fitting using LCModel software.^32,33^ In-house basis set was built using experimental acquisition scan parameters for MR spectral fitting and a spectral range of 0.5-4.2 ppm. Metabolites were considered as reliable when Cramér-Rao lower bounds (CRLBs) were ≤30% for 2HG, glutamate, glutamine, gamma-aminobutyric acid, and citrate and ≤10% for total choline (phosphocholine, glycerophosphocholine), total creatine (creatine, phosphocreatine), N-acetylaspartate, and glycine. Metabolite levels were expressed in institutional millimolar units, as well as relative to total creatine. CRLBs serve as an indicator of uncertainty in the measurement of the 2HG peak. Generally, if the CRLB exceeds the range of 50%-999%, the measurement is considered unreliable and is treated as a nondetectable 2HG (quantitative measurement equivalent to 0.0 mM concentration).

For each patient, the earliest magnetic resonance imaging with MRS was assessed for T2-FLAIR mismatch sign (T2 bright, FLAIR dark). The sign has been described as highly specific for IDH-mutant astrocytoma, although sensitivity is only moderate.^34-36^ Imaging was independently reviewed by two experienced neuroradiologists with disagreements resolved by consensus.

Enhancing and nonenhancing volumes in cubic centimeter were determined using a US Food and Drug Administration (FDA)–approved artificial intelligence-driven automated segmentation software (Neosoma Glioma v2.8.1.0, Neosoma Inc, Groton, MA) with manual corrections as needed by an expert neuroradiologist (with >25 years’ experience).

### Genetic Sequencing

Sequencing of both tissue and CSF cfDNA was performed via Memorial Sloan Kettering–Integrated Mutation Profiling of Actionable Cancer Targets (MSK-IMPACT), a Clinical Laboratory Improvement Amendments–certified, FDA-authorized next-generation sequencing assay.^29,37^ For patients who underwent biopsy, tumor DNA was extracted from formalin-fixed, paraffin-embedded tissue. For CSF sampling, CSF was collected in cfDNA BCT tubes (STRECK, 218962) and extracted. In both cases, normal DNA was extracted from mononuclear cells from patient-matched peripheral blood. The MSK-IMPACT assay covers protein-coding exons of cancer-associated genes, including *IDH1* and *IDH2*. For patients in whom MSK-IMPACT failed to identify an *IDH1/2* mutation although suspicion remained high, the banked cfDNA was reflexed to targeted testing for *IDH1/2* mutation by droplet digital polymerase chain reaction (ddPCR).

## RESULTS

### Study Cohort

Twenty-one patients with IDH-mutant brainstem gliomas were identified. The median age at diagnosis was 26 (range, 6-59) years and 57% were male. Seventy-one percent of the patients identified as White and three patients (14%) were Hispanic. All tumors with a biopsy were astrocytic (Table 1). An *IDH1/2* mutation was identified by immunohistochemistry or sequencing in 20 (95%) of 21 patients. The remaining patient was determined to be IDH-mutant by MRS alone.

**TABLE 1.** Patient Demographics and Tumor Characteristics

Patient No.	Age	Sex	Race	Ethnicity	Biopsy With IDH Mutation	Pathology	2HG Peak on MRS	IDH Mutation in CSF cfDNA	Mutation
1	44	M	White	Not Hispanic	Positive	Astrocytoma	Negative	Not available	IDH1-R132C
2	33	M	White	Not Hispanic	Positive	Astrocytoma, WHO grade 2	Positive	Not available	IDH1-R132S
3	24	W	White	Not Hispanic	Positive	High grade astrocytoma	Positive	Not available	IDH1-R132C
4	32	M	Other	Hispanic	Positive	Astrocytoma	Not available	Positive	IDH1-R132H
5	37	W	White	Not Hispanic	Positive	Astrocytoma	Negative	Not available	IDH1-R132S
6	26	W	White	Not Hispanic	Positive	Astrocytoma, WHO grade 2	Positive	Not available	IDH1-R132H
7	22	M	White	Not Hispanic	Positive	Astrocytoma	Positive	Not available	IDH1-R132H
8	37	W	White	Hispanic	Not available		Positive	Negative	Not available
9	25	W	White	Not Hispanic	Positive	High-grade astrocytoma	Not available	Not available	IDH1-R132C
10	29	M	White	Not Hispanic	Positive	Astrocytoma	Positive	Negative	IDH1-R132G
11	26	M	Black	Not Hispanic	Positive	High-grade astrocytoma	Not available	Not available	IDH1-R132S
12	23	M	Asian	Not Hispanic	Positive	Astrocytoma	Positive	Not available	IDH1-R132C
13	41	M	White	Not Hispanic	Not available		Positive	Positive^a^	IDH1-R132^b^
14	27	W	Asian	Not Hispanic	Positive	Astrocytoma	Not available	Not available	IDH1-R132C
15	5	W	Unknown	Unknown	Positive	Astrocytoma, WHO grade III	Not available	Not available	IDH1-R132H
16	35	M	White	Not Hispanic	Positive	Astrocytoma, WHO grade 2	Not available	Not available	IDH1-R132G
17	29	W	White	Not Hispanic	Not available		Positive	Positive^a^	IDH1-R132^b^
18	11	M	Arab	Not Hispanic	Not available		Not available	Positive	IDH2-R172G
19	24	W	White	Unknown	Positive	Astrocytoma	Negative	Not available	IDH1-R132C
20	22	M	White	Hispanic	Positive	Astrocytoma, WHO grade 2	Not available	Not available	IDH1-R132C
21	59	M	White	Not Hispanic	Positive	Astrocytoma, WHO grade 2	Positive	Negative	IDH2-R172S

NOTE. Age refers to age at diagnosis. Reported is the status of 2HG on the first available MRS scan (MRS from patients 4 and 20 was designated not available due to poor spectral quality).

Abbreviations: 2HG, 2-hydroxyglutarate; CSF, cerebrospinal fluid; ddPCR, droplet digital polymerase chain reaction; IDH, isocitrate dehydrogenase; MRS, magnetic resonance spectroscopy; MSK-IMPACT, Memorial Sloan Kettering–Integrated Mutation Profiling of Actionable Cancer Targets.

^a^ Identified by ddPCR (*IDH1/2* mutations in CSF were identified by MSK-IMPACT unless otherwise specified).

^b^ IDH1-R132 mutation detected by ddPCR, exact residue was not identified.

**FIG 1.**
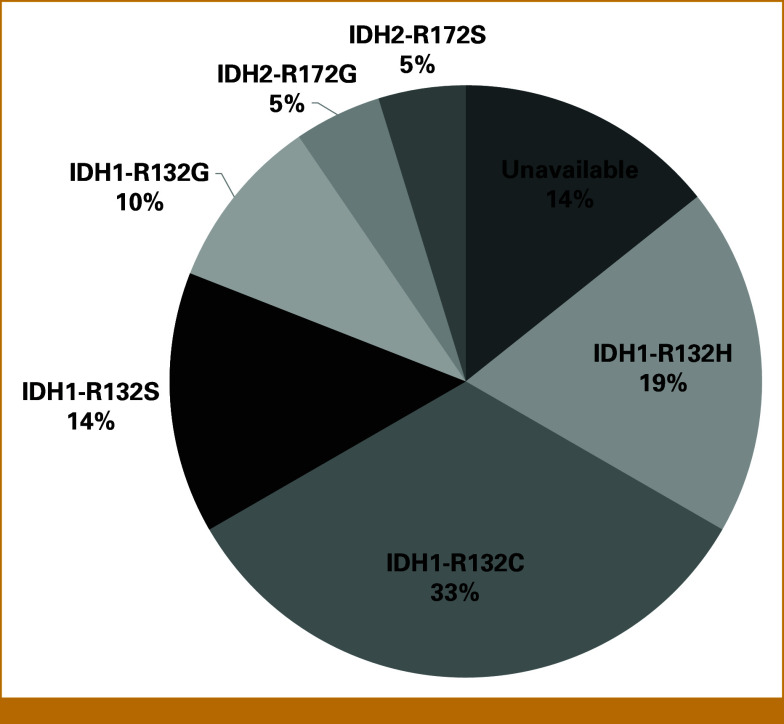
The *IDH1/2* mutations identified in this cohort brainstem gliomas. IDH1/2, isocitrate dehydrogenase 1/2.

Seventeen patients were identified by biopsy and four by noninvasive methods alone. Of the four patients diagnosed noninvasively, one patient was identified by MRS alone, one patient was identified by cfDNA in the CSF alone, and two patients had both a 2HG peak on MRS and an *IDH1/2* mutation in the CSF. Four patients had an *IDH1* R132H mutation, 12 patients had noncanonical *IDH1* mutations (*IDH1* R132C = 7, R132S = 3, *IDH1* R132G = 2), and two patients had CSF cfDNA sequencing that identified an *IDH1* R132 mutation, but the amino acid substitution could not be determined with certainty (Table 1, Fig 1). *IDH2 R172G* and *IDH2 R172S* mutations were identified in one patient each.

**FIG 2.**
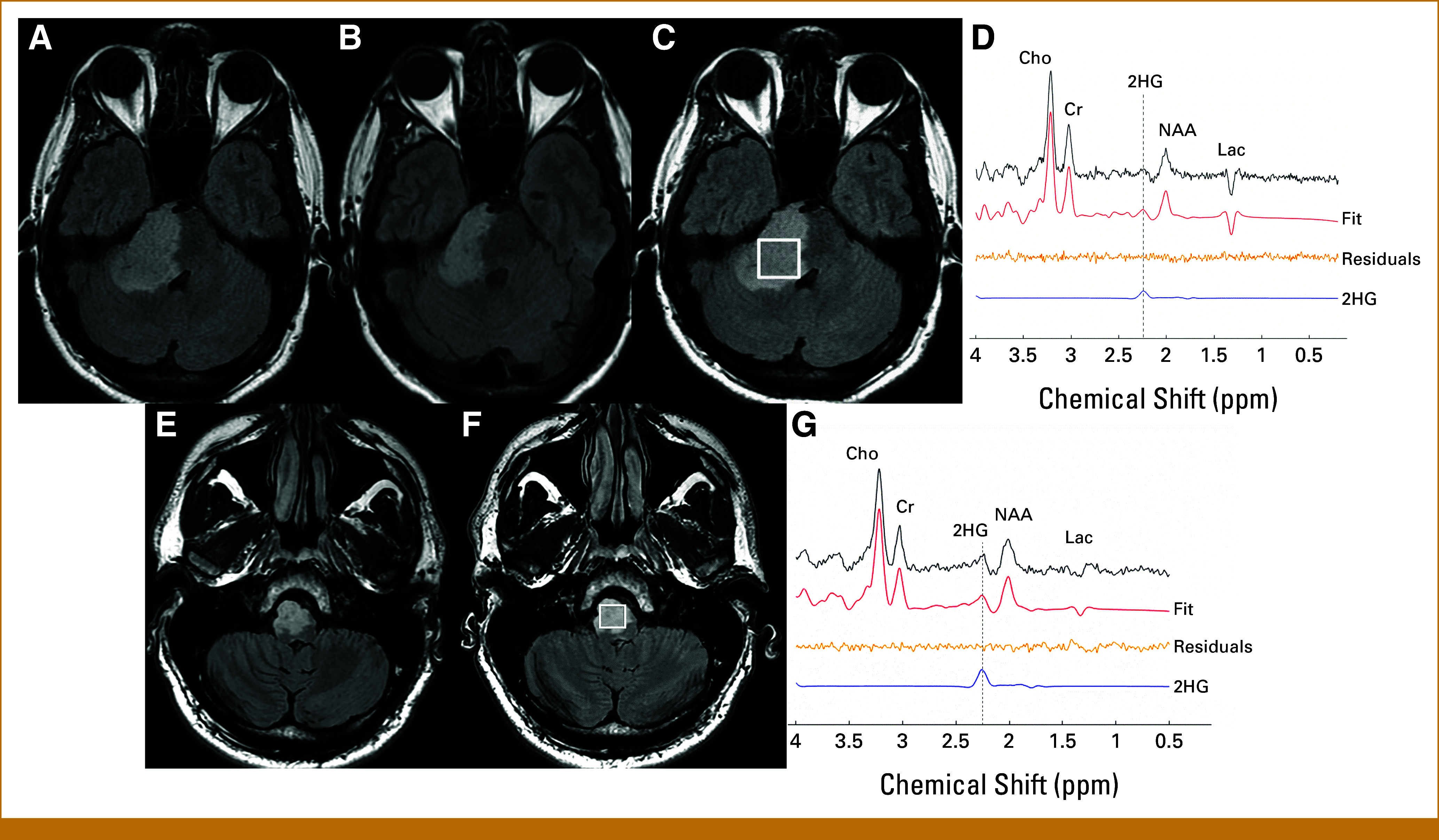
Magnetic resonance imaging with spectroscopy. For patient 13, axial T2 FLAIR before concurrent radiation and TMZ (A) and 9 months after completion of adjuvant TMZ (B); MRS voxel (C) and the MR spectrum (D). For patient 21, pretreatment axial T2 FLAIR (E), MRS voxel (F), and the MR spectrum (G). 2HG, 2-hydroxyglutarate; MRS, magnetic resonance spectroscopy; NAA, n-acetylaspartate; TMZ, temozolomide.

All patients had diffuse, expansile tumors involving the pons, either involving or abutting the brachium pontis (Table 2, Fig 2). Nine (43%) of 21 tumors involved the midbrain, 13 (62%) of 21 involved the medulla, seven (33%) of 21 involved the cerebellum, and three (14%) of 21 involved the cervical spinal cord at the time of diagnosis. Seventeen were exclusively nonenhancing, two had an enhancing component, and for two patients the presence of enhancement is unknown.

**TABLE 2.** Anatomic Distribution of Tumors and Radiographic Characteristics

Patient No.	Midbrain	Pons	Medulla	MCP	Cerebellum	C-Spine	Enhancement at Diagnosis	T2-FLAIR Mismatch
1		X		X	X		N	Absent
2		X		X	X		N	Absent
3		X	X	X			Y	Absent
4	X	X	X				N	Absent
5	X	X	X				N	Absent
6		X		X	X		N	Present
7	X	X	X	X	X		N	Absent
8	X	X	X	X			N	Absent
9		X	X			X	N	Absent
10		X	X				N	Absent
11		X	X			X	N	Absent
12		X		X	X		N	Absent
13	X	X		X	X		N	Absent
14	X	X	X	X			Unknown	Absent
15	X	X					N	Absent
16		X	X	X			N	Absent
17		X	X	X			Unknown	Absent
18	X	X					Y	Absent
19		X	X				N	Absent
20	X	X		X	X		N	Absent
21		X	X	X		X	N	Absent

Abbreviations: C-spine, cervical spinal cord; MCP, middle cerebellar peduncle.

Two radiologists reviewed the imaging for T2-FLAIR mismatch and only one patient had this imaging finding (Table 2), which has been shown to be specific for an *IDH1/2* mutation in supratentorial IDH-mutant gliomas.^11,38^

### MR Spectroscopy

Fifteen (71%) of 21 patients underwent MRS and were evaluated for a 2HG peak (Table 1, Fig 2). Excluding patient 8 without a confirmed *IDH1/2* mutation, the sensitivity of the first MRS at detecting 2HG was 64.3% (nine of 14 patients with a confirmed *IDH1/2* mutation had a 2HG peak, 95% CI, 35.1 to 87.2). Appendix Table A1 includes the MRS voxel size, 2HG concentrations, corresponding CRLBs, and enhancing and nonenhancing tumor volumes. Of the 15 patients, eight underwent their first MRS before initiation of treatment and seven underwent MRS after. Of the eight patients who had a pretreatment MRS, seven (87.5%) of eight had a 2HG peak. The sensitivity of MRS for detecting 2HG on a MRS performed before radiotherapy in patients with confirmed *IDH1/2* mutation was 85.7% (six of seven patients with a confirmed *IDH1/2* mutation had a 2HG peak, 95% CI, 42.1 to 99.6). The only patient in whom a 2HG peak was not identified (patient 5) had low signal-to-noise ratio, which may have contributed to the absence of a measurable 2HG peak. Among these eight patients, four underwent MRS both before and after radiation. Only one patient maintained a 2HG peak, which was ultimately lost 4 months after radiation therapy. In concordance, patient 7 was under surveillance with MRS for 3 years before tumor-directed intervention and had six MRS during this time, all of which had a 2HG peak. However, the 2HG peak was no longer detectable 5 months after radiation.

Seven patients (a total of 21 MRS studies) received their first MRS after radiation and TMZ. Of these, three (42%) of seven patients had a 2HG peak. The two patients with poor-quality data (patients 4 and 20) were in this group.

### CSF Evaluation

Seven patients had next-generation sequencing of CSF cfDNA (Table 1). Four patients (57%) had an *IDH1/2* mutation on CSF cfDNA. MSK-IMPACT identified the *IDH1/2* mutation for two of the four patients. For the remaining two patients, an *IDH1/2* mutation was identified upon reflex to ddPCR after MSK-IMPACT failed to demonstrate a mutation.

### Clinical Course

The patients were followed for a median of 37.7 months after completion of radiation (range, 2.5-87.9). None of the patients underwent debulking. Twenty (95%) of 21 patients underwent radiation as first-line treatment, of whom 14 (70%) of 20 received concurrent TMZ and 14 (70%) of 20 received adjuvant TMZ (Table 3). Patient 21 was planned to receive an IDH inhibitor upfront due to increased risk of radiation in the setting of multiple sclerosis. The median OS was 90.4 months and the median progression-free survival was 57.6 months. Seventeen patients had baseline imaging available before radiation. The best response to radiation, as per RANO LGG criteria, was partial response in seven, minor response in six, and stable disease in four patients.^30^ The objective response rate (complete response, partial response, and minor response) was 76%.

**TABLE 3.** Clinical Course

Patient No.	Treatment	Length of Follow-Up (months)	Survival	PFS (months)	OS (months)
1	RT/TMZ +12c adjuvant TMZ	64.38	Living	71.84	71.842
2	Proton RT/TMZ +12c adjuvant TMZ → ivosidenib	35.89	Living	169.90	169.901
3	RT/TMZ +12c adjuvant TMZ → ivosidenib	39.47	Living	40.43	46.349
4	RT/TMZ +12c TMZ → metronomic TMZ+ ivosidenib → ivosidenib alone	51.45	Living	32.20	58.586
5	RT/TMZ +12c adjuvant TMZ	65.86	Living	119.90	119.901
6	RT/TMZ +12c adjuvant TMZ	50.86	Living	58.06	58.059
7	Proton RT → bevacizumab → adjuvant TMZ (4C)+ bevacizumab	33.45	Living	32.47	76.842
8	RT/TMZ → 18C adjuvant TMZ →Ivosidenib → CCNU + bevacizumab → re-RT/TMZ → CCNU + TMZ	82.20	Deceased	59.54	88.289
9	RT → re-RT + TMZ + bevacizumab → carboplatin + bevacizumab	87.80	Deceased	71.45	90.164
10	RT/TMZ → ivosidenib → olaparib + TMZ → bevacizumab + BCNU (1c), then TMZ (1c) → partial re-RT	87.89	Deceased	57.60	90.395
11	RT to brainstem → RT to C spine → bevacizumab → bevacizumab + BCNU	39.41	Deceased	19.93	41.316
12	RT/TMZ + ongoing TMZ	6.68	Living	11.58	11.579
13	RT/TMZ → 12c adjuvant TMZ	18.26	Living	27.76	27.763
14	RT →bevacizumab → carmustine → resection → TMZ cycles	55.56	Living	53.03	67.566
15	RT/TMZ + TMZ → bevacizumab + TMZ (ongoing)	5.07	Living	7.14	7.138
16	RT/TMZ + ongoing TMZ	9.28	Living	15.43	15.428
17	RT/TMZ + ongoing TMZ	2.53	Living	15.82	15.822
18	RT alone → bevacizumab	5.30	Living	11.45	11.447
19	RT/TMZ +24c adjuvant TMZ	33.55	Living	41.51	41.513
20	RT → 12c adjuvant TMZ → PCV	35.46	Living	31.74	37.566
21	Planned for vorasidenib	NA	Living	57.89	57.895

NOTE. Treatments, length of follow-up, and survival status. Length of follow-up is calculated from the time of completion of radiation, except for patient 21 who is yet to be treated. PFS and OS are calculated from the first known abnormal MRI brain.

Abbreviations: c, cycle; MRI, magnetic resonance imaging; NA, not available; OS, overall survival; PFS, progression-free survival; RT, radiation; TMZ, temozolomide.

Five patients in our cohort were treated with the IDH inhibitor, ivosidenib. Three received it at disease progression, with disease control for a mean of 17.4 months. Two of these five were stable on ivosidenib at the time of data review, and three of these five had a positive 2HG peak that was no longer detectable after initiation of the IDH inhibitor. Two additional patients were treated with ivosidenib, but did not undergo MRS after its initiation.

## DISCUSSION

Identification of *IDH1/2* mutations in brainstem gliomas has significant treatment and prognostic implications. A stereotactic biopsy for histopathologic and molecular evaluation is indicated but is higher risk and may be inconclusive.^13,27,28^

As in previous cohorts, this study identified a high percentage of noncanonical *IDH1/2* mutations (67%).^4,11,13^ The preponderance of noncanonical mutations underscores the importance of obtaining sufficient tissue for sequencing, which is more challenging in the brainstem. Additional tools are needed to identify the presence of an *IDH1/2* mutation. This study demonstrates that MRS and CSF cfDNA are useful adjuncts for increasing diagnostic yield.

Our data show that MRS is sensitive for identification of 2HG when performed before radiation, which is consistent with the findings of Iwahashi et al.^11^ Although false positives are possible, among our database of patients with brainstem glioma who underwent MRS, none of our patients with a 2HG peak were determined to be IDH-wildtype.

Quantification of 2HG by MRS is possible using widely available 3-Tesla clinical scanners. By developing this capability, referral centers caring for patients with brainstem glioma can improve identification of *IDH1/2* mutations allowing for targeted therapy. At this time, access to 2HG quantification by MRS is limited to the largest centers because it requires advanced acquisition methods (eg, PRESS or MEGA-PRESS with optimized coils), specialized expertise in protocol optimization, spectral fitting, and interpretation. A barrier to wider adoption of this investigation technique is its investigational designation by the Centers for Medicare and Medicaid Services, which limits reimbursement.

Compared with 2HG quantification by MRS, CSF cfDNA sequencing is potentially more accessible via a reference laboratory and should be considered as a confirmatory test when tissue analysis is unavailable. Of the 21 patients with IDH-mutant gliomas, four patients were diagnosed without biopsy: two patient had a 2HG peak on MRS and an *IDH1/2* mutation on CSF cfDNA sequencing, one patient was positive by CSF cfDNA only, and one patient was inferred to be IDH-mutant based on the presence of a 2HG peak of 9.95 mM with a reliable CRLB of 11%. For this final patient without molecular confirmation, the radiographic appearance, dramatic radiographic response to treatment, and prolonged OS (>7 years) support that the tumor is IDH-mutant.

Treatment with radiation and chemotherapy appears to decrease the sensitivity of MRS. Of the four patients who underwent MRS before and after RT, three lost their 2HG peak after treatment. Andronesi et al^39^ identified a similar pattern in their longitudinal cohort of 25 patients with supratentorial IDH-mutant tumors, wherein a 48% decrease in 2HG was observed following treatment. Likewise, three patients of this study's cohort received ivosidenib and were subsequently monitored longitudinally by MRS. All three lost their 2HG peak, presumably due to the drug.

Although CSF sequencing is useful as a confirmatory test, its utility is limited by its lower sensitivity compared with 2HG quantification by MRS—only 57% of our cohort who underwent sequencing of CSF cfDNA had an *IDH1/2* mutation. Even when *IDH1/2* mutation testing is performed by ddPCR, CSF cfDNA sequencing may result in a false negative, as with patient 21. For this patient, a biopsy was required to identify the *IDH2* R172S mutation. Reflecting this result, in a separate cohort of gliomas tested with MSK-IMPACT, Miller et al identified tumor-derived cell-free DNA in the CSF of about 50% of patients with enrichment observed when disease was abutting the ventricular system or cortical surfaces.^29^

The age distribution of this study's cohort is consistent with previously described cohorts of IDH-mutant brainstem gliomas and is less biased by surgery, resulting in lower rates of enhancement and cerebellar involvement.^4,8,10-13,40^ Other cohorts reported rates of enhancement as high as 40%^4,9,14,17^ and a higher percentage of tumors that were exclusively localized to the cerebellum, where surgery is less morbid.

We show that IDH-mutant brainstem gliomas have a distinct radiographic appearance, characterized by diffuse, expansile tumors that either involve or abut the brachium pontis. These predominantly nonenhancing tumors largely lacked T2-FLAIR mismatch, which is specific for IDH-mutant supratentorial astrocytoma—consistent with other cohorts.^13^ All patients in this study who received treatment were radiated, of which 70% received concurrent and 70% received adjuvant TMZ. The median OS of 90.4 months is consistent with the literature reporting survival of up to 128 months (Appendix Table A2).^4,5,8,10-13,15,17,18^ In some publications, survival calculations are confounded by factors such as surgical complications^8,11^; as such, there is significant heterogeneity in the median survival.

This study is limited by cohort size, although the data sets reviewed were extensive. Our patients were selected by their providers to undergo MRS, which is not currently standard of care; it is unknown whether tumor size or socioeconomic status influenced patient selection. Future studies will need to perform MRS on a larger, prospective cohort of patients. In the changing landscape of glioma management, therapy is contingent on whether the tumor is astrocytic versus oligodendroglial, as well as tumor grade. Although identification of an *IDH1/2* mutation alone does not provide this information, the information from MRS, especially when supported by CSF cfDNA, remains prognostically and therapeutically useful.

In conclusion, MRS and CSF cfDNA sequencing are promising, noninvasive diagnostic techniques for the identification of *IDH1/2* mutations in brainstem gliomas. MRS is useful as a screening tool when performed before radiotherapy. Detection of 2HG using clinical MR scanners and detection of *IDH1/2* mutations by CSF cfDNA sequencing are useful as an adjunct to biopsy for identifying the patient population who would benefit from IDH-directed treatment.

## APPENDIX

**TABLE A1.** 2HG MRS for the First MRS Available

Patient No.	2HG Peak	MRS Voxel Size, mL	2HG Concentration, mM	Detectable 2HG Peak	Enhancing Volume, cm^3^	Nonenhancing Volume, cm^3^
1	No	8	0	No	1.8	8.5
2	Yes	6.5	3	Yes	0	8.1
3	Yes	2.8	8.2	Yes	6.9	17.2
5	No	1.3	0	No	0	12.2
6	Yes	8	9.1	Yes	0	59.1
7	Yes	8	5.822	Yes	0	45.6
8	Yes	5.7	8.94	Yes	0	22.8
10	Yes	5.8	5.419	Yes	0	8.3
12	Yes	10.1	4.4	Yes	0	44.4
13	Yes	8	5.8	Yes	0	22.9
17	Yes	5.3	8.1	Yes	0	9.6
19	No	7	0	No	0	2.5
21	Yes	5.1	15.5	Yes	0	7.2

Abbreviations: 2HG, 2-hydroxyglutaratespectroscopy; MRS, magnetic resonance spectroscopy.

**TABLE A2.** Literature Review of Infratentorial IDH-Mutant Gliomas

Authors	Year	No. of Patients	Confirmed IDH Mutant (n)	OS (median unless otherwise stated)
Ellezam et al^16^	2012	44	3	Survival not classified by presence of IDH mutation
Zhang et al^3^	2014	33	8	Not calculated
Theeler et al^14^	2015	143	3	2 patients stable at 7 and 14 months, 1 patient expired at 12 months
Banan et al^4^	2020	42	42	43.5 months
Chang et al^40^	2021	3	3	1.5-5 years, 1 patient deceased at 62 months
Zhou et al^8^	2022	5	5	3-28 months f/u (1 patient expired due to brainstem hemorrhage)
Pan et al^13^	2023	22	22	124.8 months
Iwahashi et al^11^	2023	10	4	Could not be estimated
Oki et al^12^	2024	12	4	85.2 months
De Simone et al^10^ (systematic review)	2025	19	19	Not calculated

Abbreviations: f/u, follow-up; IDH, isocitrate dehydrogenase; OS, overall survival.
